# The evaluation of DiGeorge syndrome gene deletion using molecular cytogenetic techniques

**DOI:** 10.1186/1471-2164-15-S2-P30

**Published:** 2014-04-02

**Authors:** Abeer A Bahamat, Sahira A Lary, Abdul Ali Peer Zada, Mohammed H AL-Qahtani

**Affiliations:** 1 Diagnostic Genomic Medicine Unit (DGMU), King Abdulaziz University, Jeddah, KSA; 2 Biochemistry Department, Collage of Science, King Abdulaziz University, Jeddah, KSA; 3 Pathology and Clinical Laboratory Medicine, King Fahad Medical City, Riyad, KSA; 4 Centre of Excellence in Genomic Medicine Research, King Abdulaziz University, Jeddah, KSA

## Background

Di-George Syndrome (DGS) is known as 22q11.2 deletion syndrome. It is a genetic disorder that is being recognized with increasing frequency with a documented incidence of approximately 1 in 4000 and is the most common human deletion syndrome, typically present early in life and is rarely appearing in adult patients (1). Micro-deletion of chromosome 22q11.2 is one of the most clinically variable syndromes, with more than 180 features associated with the deletion. The syndrome is caused by genetic deletions (loss of a small part of the genetic material) found on one of the two 22nd chromosomes (2). Very rarely, patients with similar clinical features may have deletions on the chromosome 10. An accurate diagnosis is needed for the proper management of affected cases. Diagnosis relies on conventional cytogenetic and Fluorescent In Situ Hybridization (FISH) techniques. The newly developed technique, array Comparative Genomic Hybridization (aCGH), allows for detection of minor deletions or duplications in the whole genome (3). The purpose of this study is to compare the effectiveness of using these techniques in detecting the deletion of chromosome 22q11.2.

## Materials and methods

The study included 30 suspected DGS patients depending on their symptoms, referred to the DGMU for genetic diagnosis. We used GTG-banding technique, FISH (Fluorescence in situ Hybridization) and Array-CGH (Comparative Genomic Hybridization) techniques for the detection of deletion in chromosome 22 for all patients.

## Results

**Figure 1:**
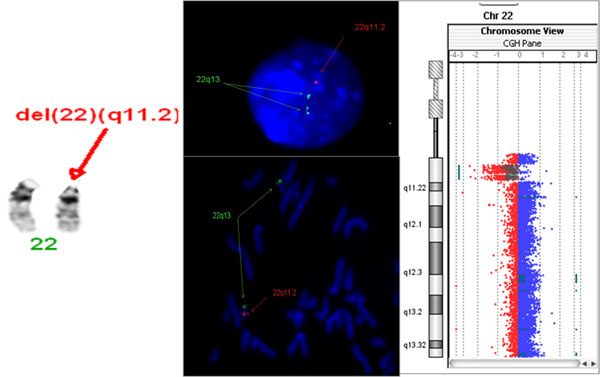
Representative image showing the detection of 22q11.2 deletion in patient #12 using three different techniques

Cytogenetic technique could detect the 22q11.2 deletion in 1/30 patients, however, other chromosomal aberrations were detected in three patients (48,XXXX/ 46,XX,del(18)(p11.2)/ 47,XX,+18). Results of FISH technique had shown the 2q11.2 deletion in 2/30 patients. The application of a-CGH technique has shown deletions in different loci on chromosome 22 in 8/30 patients as shown in Table (1) and Figure (1).

**Table 1:** Summary of the results of GTG-banding, FISH technique and aCGH technique in patients with DiGeorge syndrome

ID	Cytogenetic Results	Fish Results	Array-CGH Results
1	46,XX	46,XX.ish del(22)(q11.2q11.2)	del(22)(q11.21q11.23)

2	46,XY	no deletion	no deletion

3	46,XX	no deletion	no deletion

4	46,XY	no deletion	no deletion

5	46,XX	no deletion	del(22)(q11.21)

6	46,XX	no deletion	no deletion

7	46,XX	no deletion	del(22)(q11.23)

8	46,XY	no deletion	del(22)(q11.23)

9	46,XY	no deletion	no deletion

10	46,XX	no deletion	no deletion

11	46,XY	no deletion	no deletion

12	46,XX,del(22)(q11.2)	46,XX,del(22)(q11.2).ish del22(q11.2q11.2)	del(22)(q11.21q11.23)

13	46,XX	no deletion	no deletion

14	46,XX	no deletion	no deletion

15	46,XX	no deletion	no deletion

16	46,XY	no deletion	del(22)(q11.23)

17	48,XXXX	no deletion	no deletion

18	46,XX	no deletion	no deletion

20	46,XX	no deletion	no deletion

21	46,XY	no deletion	no deletion

22	46,XX,del(18)(p11.2)	no deletion	del(22)(q11.23)

24	46,XY	no deletion	no deletion

25	46,XY	no deletion	no deletion

26	46,XX	no deletion	no deletion

27	46,XY	no deletion	del(22)(q11.23)

28	47,XX,+18	no deletion	no deletion

29	46,XX	no deletion	no deletion

30	46,XX	no deletion	no deletion

**Total**	**1**	**2**	**8**

**%**	**3.6%**	**7.1%**	**28.6%**

## Conclusions

Array-CGH technique could detect a larger number of genome deletions or duplications in affected patients compared to FISH and cytogenetic analysis. Array-CGH is a highly sensitive technique because it depends on the scanning of the whole genome in each patient; therefore any other cryptic chromosomal aberration either a gain or loss can be accurately detected. Cytogenetic G-banding and High – resolution banding techniques could detect other chromosome aberrations such as translocation or deletion in other chromosomes. In FISH, the probe used will enable detection of a specific region only and may not cover the entire DGS region. The limitation can be overcome to some extent by use of different probes to screen the entire gene. We, therefore conclude that array-CGH is a highly sensitive technique compared to cytogenetic and FISH in the diagnosis of DGS.
